# Young People's Response to Six Smartphone Apps for Anxiety and Depression: Focus Group Study

**DOI:** 10.2196/14385

**Published:** 2019-10-02

**Authors:** Sandra Garrido, Daniel Cheers, Katherine Boydell, Quang Vinh Nguyen, Emery Schubert, Laura Dunne, Tanya Meade

**Affiliations:** 1 MARCS Institute for Brain, Behaviour & Development Western Sydney Univerity Penrith Australia; 2 Catholic Care Sydney Australia; 3 Black Dog Institute Randwick Australia; 4 University of New South Wales Kensington Australia; 5 Western Sydney University Parramatta Australia; 6 Western Sydney University Milperra Australia

**Keywords:** depression, adolescent, smartphone, mobile phone, mental health

## Abstract

**Background:**

Suicide is one of the most frequent causes of death in young people worldwide. Depression lies at the root of this issue, a condition that has a significant negative impact on the lives of those who experience it and on society more generally. However, 80% of affected young people do not obtain professional help for depression and other mental health issues. Therefore, a key challenge is to find innovative and appealing ways to engage young people in learning to manage their mental health. Research suggests that young people prefer to access anonymous Web-based programs rather than get face-to-face help, which has led to the development of numerous smartphone apps. However, the evidence indicates that not all of these apps are effective in engaging the interest of young people who are most in need of help.

**Objective:**

The study aimed to investigate young people’s response to six currently available smartphone apps for mental health and to identify features that young people like and dislike in such apps.

**Methods:**

Focus groups were conducted with 23 young people aged 13 to 25 years in which they viewed and used six smartphone apps for mental health. A general inductive approach following a realist paradigm guided data analysis.

**Results:**

The results revealed that young people value autonomy and the opportunity to personalize experiences with these apps above other things. Finding a balance between simplicity and informativeness is also an important factor.

**Conclusions:**

App developers need to consider using participant-design frameworks to ensure that smartphone apps are providing what young people want in a mental health app. Solutions to the need for personalization and increasing user engagement are also crucially needed.

## Introduction

### Background

Depression lies at the heart of what is now the second most prevalent cause of death among young people aged 15 to 29 years across the globe: suicide [1]. The reported incidence rates of major depressive disorder among young people are as high as 8% in Australia [2] and 11% in the United States [3]. However, many more young people remain undiagnosed or experience depression at subclinical levels, suggesting that the number affected is actually much higher [4].

Early intervention can reduce the duration and impact of depression, as well as reducing the chances that it will become a lifelong disability [5]. Despite this, only around 20% of affected youths obtain professional help [2]. This likely occurs for several reasons including the lack of motivation that often accompanies depression [6], a lack of understanding and knowledge about depression [7], and the stigma associated with mental illness [8]. There is thus a crucial need to find ways to engage young people with professional mental health services and in learning about mental health.

Multiple studies have demonstrated that young people prefer to access the relative anonymity of Web-based mental health resources rather than obtain face-to-face help [9,10]. The internet is one of the top sources of help that young people report seeking for mental health issues [11]. Smartphone apps are increasingly of interest in health contexts because they have the potential to provide both anonymity and accessibility, given the widespread usage of mobile phones by young people [12], with several systematic reviews reporting positive effects of usage on mental health across age groups [13,14].

Nevertheless, a recent systematic review [15] demonstrated that trial attrition rates in studies investigating the effectiveness of digital mental health interventions in young people such as smartphone apps can be as high as 70%. Even trial participants who complete the studies frequently engage minimally with the intervention throughout the duration of the study, particularly where the programs are completed unsupervised in the participant’s own time. These high dropout rates suggest that while these interventions may be *effective* in reducing symptoms of depression, they often fail to *engage* young people. In particular, the review demonstrated that the numerous digital interventions that rely on educational modules to communicate about mental health are unappealing to young users. Participants in some of the reviewed studies described such learning modules as “tiring” [16] or “tedious” [17]. Another study of apps for various health conditions including participants from multiple age groups similarly demonstrated that approximately 26% are discarded after a single use [18].

It is thus important to identify the features of mobile apps for mental health that young people find most appealing to inform future intervention design. Research across a broad range of ages suggest that features such as privacy and security of personal information are often of concern to users of mental health apps [19]. In apps relating to general health management, young people report that the *look and feel* are important factors in usage [20]. Studies have demonstrated that young people appreciate design features in mental health apps that are engaging and easy-to-use and are more motivated to use apps that fit these criteria [21]. One study explored the response of young people to a range of health-related apps in focus groups, identifying other important design criteria [22]. However, few studies have taken a similar approach in relation to mental health apps by directly investigating the response of young people to currently available apps, with a view to determining the appeal of various features to this specific population. The aim of this study, therefore, was to explore young people’s perspectives about the usability of six currently available smartphone apps for mental health to determine features that will increase appeal and engagement with future interventions.

### Research Questions

The following two research questions were asked:

1. What opinions do young people express about the usability and appeal of six currently available mental health smartphone apps?
2. Which features of these apps are most appealing or unappealing to young people and why?

## Methods

### Overview

The research question lent itself to a general inductive approach as described by Thomas [23]. This approach is commonly found in health and social sciences research and evaluation and allows detailed readings of the data to inform analysis rather than be shaped by previous hypotheses or focused on theory generation as in other inductive approaches such as grounded theory [24]. This approach was considered desirable in this context because the authors wanted the findings to be generated from the data itself rather than a priori knowledge but did not intend to develop a theory.

A general inductive approach follows a realist paradigm which takes a middle ground between constructivism and positivism, taking the view that *reality* is something that can be measured and defined independently of how we perceive it, while valuing the varying perspectives of participants [25]. Such an approach was thought to be appropriate in this study owing to the need to explore the diversity of responses to the topic under consideration, while developing an idea of the overall *reality* of user response that app developers must consider.

Focus groups were selected as the method of data collection because of the potential for interactive discussions to generate valuable details about shared experiences and diverse perspectives [26]. Focus groups are a method of data collection that have been commonly used in studies evaluating electronic health (eHealth) tools [27,28].

### Participants

The sample consisted of 24 young people aged 13 to 25 years. Participants were recruited from high schools and a university in Western Sydney, Australia, and included 15 females and 8 males. The only inclusion criterion was that participants had to be between 13 and 25 years of age. Participants were excluded if they had both scores on the Depression Anxiety Stress Scale depression subscale [29] greater than 15 (indicating severe depression; range of possible scores=0-21), and current suicidal thoughts, as there was an ethical risk associated with including high-risk participants. One participant was excluded on the basis of these criteria leaving a total sample size of 23. The excluded participant was referred to local mental health services and followed up by a clinical psychologist who was a member of the research team. Participants who were university students were provided with course credit and participants who were high school students were provided with a $50 gift voucher for study involvement.

Participants were assigned to 1 of 4 groups on the basis of their preferred attendance location, but with a view to balancing genders across the groups; 2 groups consisted of youths aged 18 to 25 years who were university students, the third group contained slightly younger participants aged 14 to 19 years including both university students and high school students, and a final group consisted of a younger age range from 13 to 15 years who were high school students (see Table 1). Participants’ depression scores ranged from no depression to severe depression, with mean scores suggesting moderate levels of depression on average across the sample.

**Table 1:** Characteristics of group participants.

Group	Total (N=23)	Female (n=15)	Male (n=8)	Age range (years)	Mean age (years)	University/high school students	Depression^a,b^
1	7	5	2	18-25	20	University	1-15
2	7	5	2	18-25	20	University	1-8
3	6	4	2	14-19	16	2 university, 4 high school	2-10
4	3	1	2	13-15	14	High school	2-9

^a^Range of scores on Depression Anxiety Stress Scale depression subscale.

^b^Total range is 1-15 with a mean of 8.

### Procedures

Ethics approval was obtained from the institutional ethics board. Notices recruiting participants for a study about music, mood, and well-being were posted around the university and in high schools. Potential participants contacted the researchers to indicate interest and were screened for eligibility in a brief phone interview by a clinical member of the research team. One participant was excluded after consultation between 2 clinical members of the research team. Eligible participants were then emailed an information sheet explaining the nature and possible consequences of the study. All participants provided written consent to participate and those under the age of 16 years also provided written consent from a parent. Each group attended 2 sessions of approximately 1.5 to 2 hours each with the sessions 1 week apart. Groups were conducted on 3 different campuses of a university in Western Sydney, Australia, in a private room. Some of the participants in the focus groups were known to each other, and 2 of the participants were known to the group-moderator. However, all participants were reminded that they were free to withdraw from the study at any stage and the moderator made a deliberate effort to allow discussions to be largely group directed so as to encourage open expression from all participants.

### Materials

Participants were questioned about their use of technology in general and then were presented with information about six different apps. The six apps presented to participants had different points of focus (Table 2), but all were concerned with mood management or management of mood disturbances, specifically depression and anxiety. All had been commercially released and were available for download on the Apple App Store. Several websites making recommendations about mental health apps were reviewed and apps that were frequently mentioned were shortlisted. The final group of six apps were selected to reflect a diverse range of approaches to mental health and a variety of features and characteristics. The selected apps were: Mood Mission, Music eScape, Pacifica, Mindshift, Headspace, and What’s Up

Mood Mission is an app developed by psychologists and researchers at Monash University, in which users rate their mood and are provided with a tailored list of tasks or missions that they can undertake to improve their mood. Music eScape was designed by researchers from the Queensland University of Technology. Users swipe their finger across the screen of their device to draw a path from the mood they are currently in to the mood they would like to get to. The app then builds a playlist from the music stored on their device designed to take them to their desired mood. Pacifica is based on principles from cognitive behavioral therapy (CBT) and mindfulness and was developed by a commercial company with a team of clinical advisors. The app allows users to track their moods, set daily challenges, access peer support communities, and explore techniques for improving mental health. Mindshift is an app for anxiety developed based on principles of CBT. It allows users to record their levels of anxiety and find ways to change the thinking patterns behind their anxiety and provides guided meditations. Headspace was designed by researchers at New York University and the University of Southern California to teach meditation and mindfulness as a stress reduction technique.What’s Up? is an app by a private developer and is based on CBT principles. It includes educational modules about challenging dysfunctional thoughts and beliefs, space for diarizing thoughts and feelings, and open forums for discussion with other people.

Participants downloaded the apps to their personal devices during the focus group or were able to use it on an iPad provided by the researchers. Discussions were guided by a topic outline, but were allowed to flow naturally as participants responded, with the moderator taking particular care to draw out diverse viewpoints from the groups. Discussions in the first session considered questions relating to app usability and appeal broadly, while the second session considered participant opinions about individual app features one-by-one in more detail (see Multimedia Appendix 1).

**Table 2:** Mental health apps assessed in focus groups.

App name	Mental health focus	Developer	Tools
Mood Mission	Depression and anxiety	Monash University	Variety of personalized behavioral strategies
Music eScape	Stress/distress	Queensland University of Technology	Develops music playlists to shift from one mood to another
Pacifica	Stress and anxiety	Commercial company with clinical advisors	CBT^a^ based mood tracking; personalized behavioral strategies; psychoeducation; links to therapists; peer support
Mind Shift	Anxiety	Anxiety Disorders Association of British Columbia	CBT approach; mood tracking; psychoeducation
Headspace	Stress	New York University and University of Southern California	Mindfulness and meditation
What’s Up?	Depression	Private developer	CBT approach; psychoeducation; social forums; diary

^a^CBT: cognitive behavioral therapy.

### Data Analysis

Focus group content was transcribed verbatim and thematic analysis performed to identify the broad explicit and implicit themes within the data using an inductive analysis style [30]. Initially, open coding was used to assign 258 segments of data to 55 codes by the first author. Once open coding had been completed, this was checked and a second wave of analysis using axial coding was conducted in a collaborative process between several authors to derive a refined set of codes. Axial coding, as described by Charmaz [31], is used to discern connections between data categories and codes. Data were clustered into six higher order latent themes with subthemes, which reflected app features that appealed or did not appeal to participants, including two crosscutting themes which applied across all other themes.

Memos were also taken throughout coding to note impressions about the influence of group dynamics, as recommended by Smithson [32], particularly as groups consisted of individuals with varying degrees of experience of depression who may have different viewpoints. Memos were based on the strategies developed by Stevens [33], including looking for statements that evoked conflict and contradictions in the discussion as well as shared experiences expressed by the participants. Notes taken by the group moderator during the sessions were also referred to in analysis.

## Results

### Overview

**Figure 1:**
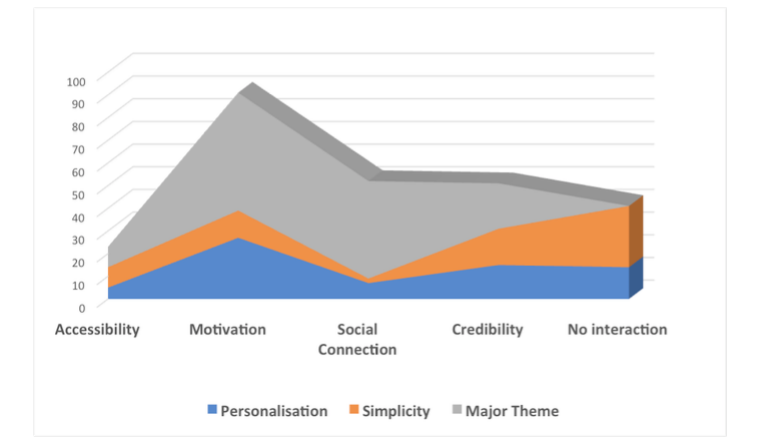
Distribution of data segments across major themes and interaction with crosscutting themes of simplicity and personalization.

Across discussions of different apps, six higher order themes were identified: accessibility, motivation, social connection, credibility, personalization, and simplicity (see Table 3). As depicted in Figure 1, the 2 crosscutting themes of simplicity and personalization permeated the discussions across all other themes. The figure shows the number of data segments allocated to each theme and the overlap with the crosscutting themes of personalization and simplicity, demonstrating that while these 2 themes were discussed in isolation to the other themes (No interaction), they also formed a part of the discussions relating to accessibility, motivation, social connection, and credibility. Simplicity held greater weight in the discussion than personalization, and motivation was the theme that had the most data segments allocated to it overall apart from the crosscutting themes. The themes and their subthemes will be discussed individually below.

### Personalization and Simplicity

As will be seen throughout discussion of other themes, many app features were perceived by participants as having both negative and positive aspects. A primary solution advocated by participants to overcome this was personalization: the ability to opt-in or opt-out of features such as the use of missions, social features, or notifications, or to tailor content and appearance to one’s own preferences and circumstances.

For example, some argued that the content itself should be customizable. For example, 1 participant said the following:

I think putting information and expecting it to work for everyone is a bad idea. Personalised is always best because what works for this person may definitely not work for this person as well.

Female, Group 1

It was suggested that:

When you first start to use the app it could give you a bunch of stuff and be like, “Okay, how do you feel about this? How do you feel about that?” and then personalize your experience based on that.

Female, Group 2

Apps that did not do that were perceived as less helpful. For example, 1 participant said in relation to Mind Shift:

The actual structure of it doesn’t assess where you’re at and then provide information on that. It’s more like here are a hundred different things and then you can try and hunt through that to find something.

Male, Group 1

**Table 3:** Higher order themes and subthemes.

Major theme	Subtheme level 1	Subtheme level 2
Personalization	Opt-in, opt-out	—^a^
	Personalized content	—
	Customizable appearance	—
	Set preferences	—
Simplicity	Minimize number of features	—
	Easy to navigate and find information quickly	—
	Information that is easy to absorb	—
	Minimalist design	—
Accessibility	Access from home	—
	Compatibility	—
	Cost	—
Motivation	Missions, rewards, objectives	Sense of achievement; requires energy
	Tracking mood	Identify patterns; discouraging if little progress
	Notifications	Useful reminders; annoying, increases guilt or anxiety
	Statistics	Understanding prevalence
Social connection	No substitute for face-to-face help	—
	Peer communication settings	Potential for inaccuracies; good to know others share your feelings; existing platforms have more content and faster response
	Anonymity	Encourages openness; a license for misuse
	Links to professional help	Live chats, hotline calls, or referrals; off-putting to some
Credibility	Original material	—
	Not dumbed down	—
	Not telling people what to do	—
	Knowing the source	—

^a^Not applicable.

Others said that having a customizable appearance, with changeable skins or color schemes would make the app feel more personal.

Simplicity was another crosscutting theme that appeared in multiple discussions. Participants across the groups were in consensus about the fact that the apps needed to be easy to access and navigate. As 1 participant said:

If it’s too difficult to use an app, I would just uninstall it. There’s just so many apps now that if it’s too difficult I’ll just find another one.

Male, Group 4

Others agreed that apps should be targeted in their focus and should not have too many features. One participant said the following:

If you have a mental health app that is also playing music, that is also telling you to exercise, there’s like a very small amount of people who will want all those three things in one app.

Female, Group 1

*What’s Up?* was one app which participants agreed contained too much information. The dominant perspective in Group 1, for example, was the following:

I wouldn’t recommend someone who’s worried to look at this app. It’s just too much information bombarded at them.

Female, Group 1

Another agreed, saying the following:

You wouldn’t want to read the whole thing because it’s just too much.

Female, Group 1

Similarly, others felt that it was important that people be able to find quick fixes easily:

When it’s very clear how to do things, that’s what’s best. Anything that requires extra thought, like if the buttons are too small so you have to squint to read them or zoom in or something or there’s way too many buttons or something. Usually that’s the stuff that’s most annoying.

Male, Group 3

Others pointed out the importance of the content being easy to absorb. As 1 participant said:

I think it’s nice to have apps that don’t require you spending a lot of time on them in one go. You can just dip in and out of them for two or three minutes at a time.

Female, Group 4

Apps that contained a lot of information or reading received a negative response from participants. For example, 1 participant said the following about Mood Mission:

If I was to download this and all of this came up, I’d be like, ‘Oh my god’ and then – because as I’m looking at it right now it’s giving me a headache.

Female, Group 1

Similarly, 1 participant said the following in relation to *Mindshift*:

There’s too much information. I feel like it tells you ‘Do this, do this, do this, do this’, but it’s not giving you suggestions on how to get to that.

Female, Group 2

Aesthetically, simplicity was important to users as well. In relation to *Music eScape*, a participant said the following:

I like that it’s very minimal as well. It’s colourful but it’s still simple

Female, Group 1

*Headspace* was another app which garnered positive comments about the design across the groups. As a participant said in relation to *Headspace*:

There’s a very specific aesthetic that I enjoy and it’s that very clean, simplistic, almost IKEA-ish look.

Female, Group 3

This opinion prompted other participants in the group to agree on the need for clarity and simplicity in both the esthetic and the content. In fact, as stated by another participant in that group, an overly complex design could add to a user’s sense of anxiety:

Some apps that can be really busy can be really anxious. Especially a mental health app, being really clean cut it’s just calming and good to look at it and it just pleases you. It kind of clears you. It’s like, “Everything’s in order and it’s great.”

Female, Group 3

### Accessibility

One perceived advantage to the use of apps was the ability to access it from home or any other place. Participants commented on how people who feel depressed find it difficult to leave the house or to speak to people in person about their problems. One participant stated as follows:

A lot of times people who are really depressed just don’t want to leave the house. They can’t be bothered filling up their Opal card [a public transport ticket used in Australia], or catching a bus is too much effort, or they panic. So, there should be a way to access those things within your home.

Female, Group 3

Compatibility was another aspect that influenced how accessible users found the apps considered in this study. It was also important to participants that apps be accessible across a wide range of devices including phones, computers, and iPads, and that they be compatible with both Android and Apple operating systems. Participants also pointed to some issues relating to accessibility with *Music eScape*. In Group 1, despite a high level of enthusiasm for the overall concept and the design, there was agreement that the app’s reliance on music stored on one’s device limited its usefulness. Consensus within the group on this issue was evidenced by the way the participants finished each other’s sentences, indicating their shared viewpoints. Several participants said that they would only use the app if it was compatible with music streaming platforms such as Spotify, but on exploring the app, the groups found that it was not and expressed the opinion that this would render it unusable for them. Participants in Group 2 similarly reached consensus about this limitation, as expressed by 1 participant:

I don’t save music to my phone.

Female, Group 2

Another prohibitive aspect of some apps that related to accessibility was cost. For example, in Group 2, participants began discussions about *Pacifica* by agreeing that they would not want to pay for a mental health app:

What’s the point when there’s other apps that could do the same thing it does but they could do it, let’s say, for no charge.

Female, Group 2

However, 1 participant disagreed with the others, stating the following:

When it comes to anyone feeling better, whether it’s going to the gym or things like that, people will pay because they’re wanting to feel better within themselves.

Female, Group 2

This led the group to agree that there might be situations in which they would be willing to pay for a mental health app, such as if it was highly reputable and if the cost was a one-off, low payment. Nevertheless, this emerged as a negative point in relation to other apps as well, such as this comment in relation to *Headspace:*

It annoyed me a bit because it’s got a lot of stuff that sounds cool but then when you click on it, it tries to make you sign up for a subscription.

Female, Group 2

### Motivation

Motivation was a theme that emerged from discussions about features found in several apps. The features under discussion were typically interactive features that required the user to engage in particular activities, such as missions, objectives and rewards, and mood tracking. Participants across the groups held varying viewpoints about whether these features would be motivating or demotivating to users and whether they would encourage them to engage more or less with the app or with activities that could benefit their mental health. The effect of notifications and statistics about other users on user motivation was also discussed. Participants argued that they could be both motivating and discouraging or could require more energy than a depressed user would have. For example, in relation to Mood Mission, several participants like the ideas of missions or objectives, stating:

I feel like achievements would be good if it was there because it kind of motivates you.

Female, Group 2

However, others stated:

It could be helpful but at the same time you wouldn’t want it to remind you constantly that you have to do this or that. What if there are days where you just can’t be bothered?

Female, Group 1

This opinion was echoed by several participants who argued that when a user is lacking in motivation and energy because of depression, the concept of missions could be overwhelming. Thus, it was suggested that it would be best to make the use of missions or objectives

Optional so that if you want it you can use it. If you don’t want to, no problem.

Male, Group 1

Similarly, in relation to mood tracking features such as in *Pacifica*, some argued that it could be helpful to be able to identify patterns:

Something cool about this app is that you can actually track the progress of what’s going on. Which can be a good thing because if someone really wants to feel better, for example, it could help them and motivate them even more.

Female, Group 2

However, 1 participant felt that:

Seeing the graph if you don’t see any progress, that can frustrate you.

Female, Group 1

Another said:

This is more for like elderly, or people in their forties, like that age category, because especially for younger—we don’t—I don’t know, personally I wouldn’t want to go to this app because I wouldn’t like to record my feelings and write it down.

Female Group 2

Notifications were also viewed as something that could also be motivating or demotivating and that should therefore be personalizable. Participants in Group 4, for example, shared differing perspectives on the use of notifications, with 1 female and 1 male participant agreeing that they liked reminders and notifications to keep them engaged with an app, while another male participant stated that he always opted to turn them off. Participants from other groups similarly stated:

If it’s daily it can become a chore. It just off puts people and mitigates the effects.

Male, Group 1

Another indicated that notifications could even be a source of anxiety saying:

It shouldn’t bother you. It should never pester you. It’s meant to stop anxiety, not increase it.

Female, Group 3

### Social Connection

Some of the apps considered in this study contained features that allowed social connection with others. Participants across the groups voiced a range of perspectives on this subject, pointing out both pros and cons of these features. Several participants felt that despite the convenience of being able to access support on one’s phone, this was no substitute for face-to-face contact. One participant argued:

I think all these apps, they can get unhealthy when people use them as a crutch and they get addicted to them instead of getting proper health, because their mental health will be deteriorating but they won’t really care because they’ll be like, “as long as I’m using this app I’m fine.” They won’t go out and talk to someone. They won’t get help and will just use it as a crutch.

Female, Group 1

Others felt that there were some distinct disadvantages to accessing peer support communities. Groups 2 and 3 particularly discussed this in some detail. Despite some agreement that:

When you know that someone else shares the same feelings as you, you could maybe be supporting each other.

Female, Group 2

Others in the group argued that there was the potential to be given inaccurate advice. One participant said the following:

Whoever is going on the app is going to get a lot of conflicting information.

Female, Group 2

Similar viewpoints were expressed in other groups, such as this statement:

I don’t necessarily just want to speak to ‘randos’ who are in the same position as me. I don’t really see how that would be productive.

Female, Group 3

Others felt that existing social platforms would be preferable. As 1 participant said:

You may as well go on the internet and some of the more popular sites where everyone is using it and get more responses then. Probably someone who is in your situation has already posted something related to where you are.

Male, Group 1

Similar conflicting views were shared about the anonymity of online forums. One participant said the following:

If it’s online then you’re more inclined to talk to other people because they don’t know where you are

Male, Group 1

Another said:

I definitely think it would be nice to be a bit anonymous. Especially if I’m feeling a bit sad, I don’t always want other people to know about that.

Male, Group 4

However, others argued that the anonymity could give license to some users to use online forums in an unhelpful way:

People can be spreading negative energy towards other people. When people talk about depressing stuff it can make you depressed.

Female, Group 2

As another pointed out:

I think the best place for someone who is an absolute psychopath to push people over the edge would be a mental health app where a bunch of mentally ill people are trying to get help from strangers.

Female, Group 3

The range of perspectives shared within the groups prompted suggestions from participants about how the relative benefits and drawbacks could be negotiated. Some participants felt that it would be better if the apps connected individuals with professionals. One participant said the following:

Maybe there could be this little online chat with someone who could actually offer support.

Female, Group 2

However, participants in Group 3 argued that this could also be off-putting to some:

If you’re going to connect to a psychologist or a psychiatrist then you’re not talking to a stranger. They’ve got an identity in your mind. They’re an authority type person who you’re talking to.

Male, Group 3

Another participant shared a personal anecdote that illustrated this, saying:

I went to one of these apps when I was 13 or 14 and talking to someone, and after I finished talking to them it made me afraid that they were going to find me. I just quit the app and I was never looking at this again.

Female, Group 3

Other suggestions were that social forums would need to be highly moderated or that social features should be on an opt-in, opt-out basis.

### Credibility

Participants agreed that app materials needed to be carefully worded to gain credibility with users. When materials were perceived as unoriginal or clichéd, containing too much information that was readily available elsewhere, this was off-putting to participants. For example, participants in Groups 1, 2, and 4 felt that the content of Mood Mission did not provide anything unique. As 1 participant stated:

The gist of relaxing, focusing and just breathing deeply is in almost every other meditation, so you don’t really need another one. Common knowledge basically.

Male, Group 1

Another participant from the same group similarly said about *Mindshift*:

A quick Google search would probably bring up all of this and more and you don’t have to download the app.

Male, Group 1

For others, it was the use of language that they perceived as clichéd that was unappealing. When speaking about *Headspace*, 1 participant said the following:

It can get irritating because it just falls back on the whole cliché thing. I don’t like them using euphemisms when it comes to mental health.

Female, Group 1

On the other hand, apps that took a unique approach to managing moods and mental health, such as *Music eScape*, were of interest to the participants. A number of participants across the groups liked the concept of using music to manage their mood:

I’m a music person so one hundred percent I would definitely use that.

Female, Group 1

However, users in Group 2 agreed that the lack of a clear mental health message in this app created the potential for the app to be used ineffectively. As articulated by 1 participant:

What if somebody wanted to go from happy to sad? There’s something kind of questionable about that feature even being there. It’s not really a positive mental health app—you can be happy and then go to aggressive if you wanted to. So it’s not really focused on positive mental health

Female, Group 2

Nevertheless, 1 dissenting voice in Group 2 pointed out that the more subtle approach to mental health found in this app could have appealed to people less willing to engage with content about mental health.

Others disliked that the language used in some apps could be:

A bit dumbed down, like they’re for kids.

Female, Group 4

As another participant put it:

You don’t want it to be shameful. You don’t want to be talked down to. You want to be spoken to in an encouraging way where you actually want to motivate yourself to get better.

Female, Group 3

For others, knowing that the information contained in the app was scientifically based or that the app had been developed by experts was important to credibility. Thus, it was important to participants that the language used in the app gave the user confidence in the app and its content.

## Discussion

### Principal Findings

The aim of this study was to explore the responses of young people to six currently available mental health apps and to determine the features that they found appealing and useful in those apps. This study revealed that young people have clear views about how mental health apps should work to benefit them.

The data were organized into six themes: accessibility, motivation, social connection, and credibility, and the 2 crosscutting themes of personalization and simplicity. Some of our findings, such as the importance of apps being easy to access and easy to use, are common findings in the literature about development of eHealth apps. For example, Crane et al [34], conducted think-aloud sessions and interviews with users of an app to reduce excessive alcohol consumption, and found that making the app easy to use and navigate through as well as avoiding excessive text or options were important to users. Criteria like this are found in common tools for evaluating app appeal and usability [35]. However, this study revealed some additional criteria that add to an understanding of how young people respond to mental health apps.

### Personalization

One key finding of this study was that participants valued the capacity to demonstrate autonomy, and to personalize their experiences with the apps from content to appearance. User wanted to be able to customize both the visual aspect of the app and the way they interacted with app content.

In general, trends are shifting in favor of models of personalized care and treatment strategies across multiple areas of health care [36]. At the heart of these trends are principles derived from theories such as Self-Regulation Theory, which argues that a sense of personal control and empowerment is important to motivating individuals to successfully meeting personal challenges [37]. Personalization in health care has been found to help individuals to develop greater autonomy and capacity to self-manage their well-being [38]. The literature on eHealth interventions similarly highlights the need for personalizability. For example, a content analysis of Web reviews of apps for bipolar disorder found that users commonly complained about features that did not meet the differing needs of individuals and asked for apps to be more customizable [39]. One study of user perspectives of an app to promote physical activity similarly found that personalization was an important factor in user engagement [40]. Thus, app developers need to keep in mind that a one-size-fits-all approach is unlikely to satisfy different users’ preferences and needs.

As the need to balance standardized procedures with personalization presents a challenge to developers, future research should explore the development of innovative interventions that can adapt and respond to a variety of user needs and preferences. Tailoring matrixes have been effectively used in eHealth interventions in the past to personalize content based on feedback to user responses [41], and such models provide a basis for further innovation in this area.

### Balancing Simplicity With Credibility

Another key point that emerged from this study is the need to balance informativeness and credibility with simplicity. Participants did not want to see material that was clichéd, repetitive, or unoriginal. They wanted to see scientific evidence and to know that the information had credibility. This is in contrast to some previous studies which have shown that users sometimes integrate apps into their health management without regard for the evidence-base or clinical effectiveness [39]. It may be that young users are becoming more aware of the proliferation of apps that are developed by private and corporate developers [42]. Even participants from the youngest age group in this study expressed that they did not want to be *talked down to* or to feel the app was just for kids. However, it was important that information be presented simply, with minimal amounts of text that is easy to find and absorb.

Theoretical frameworks for eHealth development such as the Technology Acceptance Model similarly highlight the importance of perceived ease of use to the user, as well as perceived usefulness in increasing user engagement [43]. Simplicity and ease of use is particularly important for app developers to consider in mental health contexts, as depression and anxiety are associated with a lack of motivation [44](2) and impaired concentration [45]. Thus, apps that contain large amounts of text or complex materials are unlikely to be engaging to young people, particularly those experiencing mental health difficulties. A key challenge for app designers, therefore, is to develop features that are perceived as effective, useful, and informative but that involve low levels of user burden.

One key point to this that was revealed in this study is that participants appreciated apps that focused on 1 feature such as meditation, and that approached mental health in a unique way such as in *Music eScape*. In fact, given the importance that music has in the daily lives of young people, with up to 18 hours/week invested in listening to it [46], music provides an avenue for engaging young people in learning about mental health that has potential for further exploration. Users in this study were particularly intrigued with this way of exploring mental health given their high level of intrinsic interest in music. Nevertheless, participants identified significant drawbacks from this app, in that it did not cater for users who do not store a variety of music on their phone nor did it help users understand how to use music effectively. Previous research has also demonstrated that not all music has a beneficial effect on mental health [47,48]. Future app development could look at building on music streaming platforms that cater to a wide variety of tastes while providing guidance to users about making helpful choices.

Game-based apps may also provide another useful tool for engaging young people in learning about mental health. Studies have demonstrated some positive effects of game-based programs [49]. However, even game-based apps tend to be primarily of interest to young people when they are already engaged in addressing their mental health (15), suggesting that more work is needed to develop apps that provide a level of interest and interaction that is sufficient to engage young people who are otherwise averse to learning about mental health. Furthermore, depression is commonly associated with internet addiction and excessive online gaming [50] and some types of games have been associated with increased depression [51]. This highlights the need for a careful approach to development of game apps for mental health, such as the work by the Games for Emotional Mental Health Lab in The Netherlands (see, eg, Schoneveld et al [52]).

### Social Features

The study further highlighted some of the pros and cons of social features in mental health apps. While young people might appreciate knowing that other people feel the same way as them and appreciate the opportunity to express their experiences in an anonymous environment, participants in this study were aware of the potential for misinformation or even abuse when seeking advice from nonprofessionals.

This illustrates a paradox that has been previously observed in Web contexts that some researchers call “the online disinhibition effect” [53], referring to the tendency to both self-disclose (benign disinhibition) and to act out (toxic disinhibition) more than usual in anonymous contexts. Other scholars have similarly concluded that social networking features in an app are a *gamble* because of the need to balance the potential for negative and positive effects [54]. Thus, peer communications in online mental health settings require careful planning to ensure that they are both safe and helpful for psychologically vulnerable individuals. Moderation of Web communications may need to be in place, and measures should be taken to preserve user confidence in anonymity when linking to professional mental health services as well.

User-centered and participatory design frameworks may be particularly important in developing solutions to the design challenges noted above [55]. Participatory design frameworks integrate young people at all stages of the research process as co-researchers and co-designers of interventions. Hagen et al [55] suggest a 6-stage approach to co-design in which young people are involved in focus groups, co-design workshops, and other studies to: (1) identify a health issue, (2) define the factors contributing to the problem, (3) position the problem in the context of current evidence, (4) develop an intervention concept, (5) create prototypes and test models, and (6) test and evaluate the resulting intervention. Co-design of mental health interventions alongside young people can do much to contribute to the appeal of such interventions and the degree to which they will be engaging and relatable to their target market. Future research and development of smartphone apps for mental health should therefore be focused on the use of evidence-based strategies as well as engaging end users throughout the development and evaluation processes.

This study was limited to some degree in the use of focus groups as a methodology. Although this methodology can be useful for drawing out multiple perspectives, there is some possibility that group dynamics in some way shaped the perspectives expressed by participants. In particular, as the groups contained people both with lived experience of depression and those without, this may have discouraged more personal expressions from participants with experience of mental illness. The fact that members of the groups were known to each other can also influence how people communicate about potentially personal topics. While the group moderator and methods of analysis attempted to take these factors into consideration, results should be read with this limitation in mind. The study is further limited by the small number of apps considered and the relatively small amounts of interaction time that participants had with each app, which may have reduced their capacity to develop a comprehensive viewpoint of app functionality. Nevertheless, this may reflect the reality of how users make decisions about app use, with studies demonstrating that young people tend to engage fleetingly with apps and to rapidly discard those that do not meet their expectations [22]. Future studies should look at a wider range of mental health apps and could consider allowing users to engage with them over longer periods of time.

### Conclusions

On the whole, this study demonstrates that mental health apps need to cater to the individuality of the users. Features to improve user experience and engagement include personalization by developing customizable content and user interfaces, as well as providing feedback and progress tracking for the individual, on an opt-in, opt-out basis. Overburdening users with a lot of reading and other content is not appealing to many young users, leading to a lack of engagement with many currently available apps. It is therefore crucial to develop concrete and action-oriented features with a fun and entertaining design to motivate millennials to learn about mental health.

In summary, we make 3 key recommendations for the future development of smartphone apps for both the treatment and diagnosis of mental health issues: (1) end users should be closely involved in all stages of the design process from problem identification to evaluation and testing, (2) app designs need to incorporate innovative ways to provide customizable content that can adapt and respond to individual user needs and preferences, (3) increase user engagement by balancing informativeness with simplicity and building on highly interactive activities that young people are already engaged in such as music listening and gaming.

## Appendix

Multimedia Appendix 1 Focus group discussion guide.

## Abbreviations

CBT: cognitive behavioral therapy
eHealth: electronic health

## References

[1] (2017). World Health Organization.

[2] Lawrence D, Johnson S, Hafekost J, de Haan KB, Sawyer M, Ainley J, Zubrick SR (2015). Australian Government Department of Health.

[3] Merikangas KR, He J, Burstein M, Swanson SA, Avenevoli S, Cui L, Benjet C, Georgiades K, Swendsen J (2010). Lifetime prevalence of mental disorders in US adolescents: results from the national comorbidity survey replication--adolescent supplement (NCS-A). J Am Acad Child Adolesc Psychiatry.

[4] Williams SZ, Chung GS, Muennig PA (2017). Undiagnosed depression: a community diagnosis. SSM Popul Health.

[5] Meade T, Dowswell E (2016). Adolescents' health-related quality of life (HRQoL) changes over time: a three year longitudinal study. Health Qual Life Outcomes.

[6] Hershenberg R, Satterthwaite TD, Daldal A, Katchmar N, Moore TM, Kable JW, Wolf DH (2016). Diminished effort on a progressive ratio task in both unipolar and bipolar depression. J Affect Disord.

[7] Coles ME, Ravid A, Gibb B, George-Denn D, Bronstein LR, McLeod S (2016). Adolescent mental health literacy: young people's knowledge of depression and social anxiety disorder. J Adolesc Health.

[8] Clement S, Schauman O, Graham T, Maggioni F, Evans-Lacko S, Bezborodovs N, Morgan C, Rüsch N, Brown JS, Thornicroft G (2015). What is the impact of mental health-related stigma on help-seeking? A systematic review of quantitative and qualitative studies. Psychol Med.

[9] Burns JM, Davenport TA, Durkin LA, Luscombe GM, Hickie IB (2010). The internet as a setting for mental health service utilisation by young people. Med J Aust.

[10] Leach LS, Christensen H, Griffiths KM, Jorm AF, Mackinnon AJ (2007). Websites as a mode of delivering mental health information: perceptions from the Australian public. Soc Psychiatry Psychiatr Epidemiol.

[11] (2016). Mission Australia.

[12] Lenhart A (2015). Pew Research Center.

[13] Firth J, Torous J, Nicholas J, Carney R, Pratap A, Rosenbaum S, Sarris J (2017). The efficacy of smartphone-based mental health interventions for depressive symptoms: a meta-analysis of randomized controlled trials. World Psychiatry.

[14] Firth J, Torous J, Nicholas J, Carney R, Rosenbaum S, Sarris J (2017). Can smartphone mental health interventions reduce symptoms of anxiety? A meta-analysis of randomized controlled trials. J Affect Disord.

[15] Garrido S, Millington C, Cheers D, Boydell K, Schugert E, Meade T, Nguyen QV (2019). What works and what doesn't work? A systematic review of digital mental health interventions for depression and anxiety in young people. Front Psychiatry.

[16] Gerrits RS, van der Zanden RA, Visscher RF, Conijn BP (2007). Master your mood online : a preventive chat group intervention for adolescents. Aus e-J Adv Ment Health.

[17] Burckhardt R, Manicavasagar V, Batterham PJ, Miller LM, Talbot E, Lum A (2015). A web-based adolescent positive psychology program in schools: randomized controlled trial. J Med Internet Res.

[18] (2019). Consumer Health Information Corporation.

[19] Thornton LK, Kay-Lambkin FJ (2018). Specific features of current and emerging mobile health apps: user views among people with and without mental health problems. Mhealth.

[20] Chan A, Kow R, Cheng JK (2017). Adolescents’ perceptions on smartphone applications (apps) for health management. J Mob Technol Med.

[21] Fuller-Tyszkiewicz M, Richardson B, Klein B, Skouteris H, Christensen H, Austin D, Castle D, Mihalopoulos C, O'Donnell R, Arulkadacham L, Shatte A, Ware A (2018). A mobile app-based intervention for depression: end-user and expert usability testing study. JMIR Ment Health.

[22] Dennison L, Morrison L, Conway G, Yardley L (2013). Opportunities and challenges for smartphone applications in supporting health behavior change: qualitative study. J Med Internet Res.

[23] Thomas DR (2016). A general inductive approach for analyzing qualitative evaluation data. Am J Eval.

[24] Strauss AL, Corbin J (1998). Basics of Qualitative Research: Techniques and Procedures for Developing Grounded Theory.

[25] Sobh R, Perry C (2006). Research design and data analysis in realism research. Eur J Mark.

[26] Krueger RA, Casey MA (1994). Focus Groups: A Practical Guide for Applied Research.

[27] Koskela T, Sandström S, Mäkinen J, Liira H (2016). User perspectives on an electronic decision-support tool performing comprehensive medication reviews - a focus group study with physicians and nurses. BMC Med Inform Decis Mak.

[28] Donnelly LS, Shaw RL, van den Akker OB (2008). eHealth as a challenge to 'expert' power: a focus group study of internet use for health information and management. J R Soc Med.

[29] Henry JD, Crawford JR (2005). The short-form version of the depression anxiety stress scales (DASS-21): construct validity and normative data in a large non-clinical sample. Br J Clin Psychol.

[30] Braun V, Clarke V, Hayfield N, Terry G, Liamputtong P (2019). Thematic analysis. Handbook of Research Methods in Health Social Sciences.

[31] Charmaz K (2006). Constructing Grounded Theory: A Practical Guide through Qualitative Analysis.

[32] Smithson J (2000). Using and analysing focus groups: limitations and possibilities. Int J Soc Res Methodol.

[33] Stevens PE (1996). Focus groups: collecting aggregate-level data to understand community health phenomena. Public Health Nurs.

[34] Crane D, Garnett C, Brown J, West R, Michie S (2017). Factors influencing usability of a smartphone app to reduce excessive alcohol consumption: think aloud and interview studies. Front Public Health.

[35] Stoyanov SR, Hides L, Kavanagh DJ, Zelenko O, Tjondronegoro D, Mani M (2015). Mobile app rating scale: a new tool for assessing the quality of health mobile apps. JMIR Mhealth Uhealth.

[36] El-Alti L, Sandman L, Munthe C (2019). Person centered care and personalized medicine: irreconcilable opposites or potential companions?. Health Care Anal.

[37] Leventhal H, Leventhal EA, Contrada RJ (1998). Self-regulation, health, and behavior: a perceptual-cognitive approach. Psychol Health.

[38] Schmittdiel J, Mosen DM, Glasgow RE, Hibbard J, Remmers C, Bellows J (2008). Patient assessment of chronic illness care (PACIC) and improved patient-centered outcomes for chronic conditions. J Gen Intern Med.

[39] Nicholas J, Fogarty AS, Boydell K, Christensen H (2017). The reviews are in: a qualitative content analysis of consumer perspectives on apps for bipolar disorder. J Med Internet Res.

[40] Tong HL, Coiera E, Laranjo L (2018). Using a mobile social networking app to promote physical activity: a qualitative study of users' perspectives. J Med Internet Res.

[41] Kassavou A, Houghton V, Edwards S, Brimicombe J, Sutton S (2019). Development and piloting of a highly tailored digital intervention to support adherence to antihypertensive medications as an adjunct to primary care consultations. BMJ Open.

[42] Nicholas J, Boydell K, Christensen H (2016). mHealth in psychiatry: time for methodological change. Evid Based Ment Health.

[43] Davis FD (1989). Perceived usefulness, perceived ease of use, and user acceptance of information technology. MIS Q.

[44] Loonen AJ, Ivanova SA (2016). Circuits regulating pleasure and happiness in major depression. Med Hypotheses.

[45] Boschloo L, van Borkulo CD, Borsboom D, Schoevers RA (2016). A prospective study on how symptoms in a network predict the onset of depression. Psychother Psychosom.

[46] Papinczak ZE, Dingle GA, Stoyanov SR, Hides L, Zelenko O (2015). Young people's uses of music for well-being. J Youth Stud.

[47] Wilhelm K, Gillis I, Schubert E, Whittle EL (2013). On a blue note: depressed peoples' reasons for listening to music. Music Med.

[48] Garrido S, Schubert E (2015). Music and people with tendencies to depression. Music Percept.

[49] Merry SN, Stasiak K, Shepherd M, Frampton C, Fleming T, Lucassen MF (2012). The effectiveness of SPARX, a computerised self help intervention for adolescents seeking help for depression: randomised controlled non-inferiority trial. Br Med J.

[50] Liu L, Yao YW, Li CR, Zhang JT, Xia CC, Lan J, Ma S, Zhou N, Fang X (2018). The comorbidity between internet gaming disorder and depression: interrelationship and neural mechanisms. Front Psychiatry.

[51] Bonnaire C, Baptista D (2019). Internet gaming disorder in male and female young adults: the role of alexithymia, depression, anxiety and gaming type. Psychiatry Res.

[52] Schoneveld EA, Malmberg M, Lichtwarck-Aschoff A, Verheijen GP, Engels RC, Granic I (2016). A neurofeedback video game (MindLight) to prevent anxiety in children: a randomized controlled trial. Comput Human Behav.

[53] Suler J (2004). The online disinhibition effect. Cyberpsychol Behav.

[54] Singleton A, Abeles P, Smith IC (2016). Online social networking and psychological experiences: the perceptions of young people with mental health difficulties. Comput Human Behav.

[55] Hagen P, Philippa C, Metcalf A, Nicholas M, Rahilly K, Swainston N (2012). Western Sydney University.

