# Supplementary material for: Young People's Response to Six Smartphone Apps for Anxiety and Depression: Focus Group Study
Source: JMIR Ment Health. 2019 Oct 2;6(10):e14385. doi: 10.2196/14385 (PMC6915797; doi:10.2196/14385)
Supplement: Multimedia Appendix 1 [file mental_v6i10e14385_app1.pdf]

Focus Group Discussion Guide

Introduction

- Facilitators introduce themselves and explain their roles
- Group members introduce themselves where not known to each other
- Explaining the purpose of the focus groups and re-iterate the points in the Participant Information Sheets

General Discussion Questions:

1. Go around the room and have people name an app that they use and a reason why they like it.
2. What particular features make mobile applications or web-sites appealing to young people?
3. How familiar are you with the apps in the list?
4. Were there any of the apps for discussion that you find particularly interesting or would be more likely to use?

Discussion about Individual Apps

1. What do you think about how this app looks?
2. Which features do you like or dislike about this app?
3. How likely would you be to use this app?
4. Draw attention to individual features of each app such as social forums, discussion boards, interactive exercises, informative text and ask what people think about these features.
